# Association between the polymorphism of IL-17A and IL-17F gene with knee osteoarthritis risk: a meta-analysis based on case-control studies

**DOI:** 10.1186/s13018-019-1495-0

**Published:** 2019-12-16

**Authors:** Feifan Lu, Pei Liu, Qidong Zhang, Weiguo Wang, Wanshou Guo

**Affiliations:** 1 China-Japan Friendship School of Clinical Medicine, Peking University, No.2 Yinghua East Street, Beijing, 100029 China; 2 Beijing University of Chinese Medicine, Yinghua East Street, Beijing, 100029 China; 3 Department of Orthopedic Surgery, Beijing Key Lab Immune-Mediated Inflammatory Diseases, China-Japan Friendship Hospital, No.2 Yinghua East Street, Beijing, ,100029 China

**Keywords:** IL-17, Polymorphisms, Knee, Osteoarthritis, Meta-analysis

## Abstract

**Background:**

Knee osteoarthritis is a joint disease which is characterized by degeneration of articular cartilage and subsequent subchondral bone changes. Polymorphisms of IL-17A/F gene were the recognized candidate genes associated with knee osteoarthritis risk although the results were conflicting. The aim of this study was to determine whether IL-17A(rs2275913) and IL-17F(rs763780) polymorphisms confer susceptibility to knee osteoarthritis.

**Method:**

Literature search was performed in PubMed, Medline, Cochrane Library, Web of science, Embase, and Google Scholar (last search was updated on June 20, 2019), and assessing this association was performed by calculating odds ratios with 95% confidence intervals. Statistical heterogeneity was quantitatively evaluated by using the *Q* statistic with its *p* value and *I*^2^ statistic.

**Result:**

Six case-control based studies were included involving IL-17A(rs2275913) (2134 cases and 2306 controls) and IL-17F(rs763780) (2134 cases and 2426 controls). The overall analysis suggested that the A allele of the rs2275913 polymorphism, and the C allele of the rs763780 polymorphism in the IL-17 gene may increase the risk of OA. However, subgroup analysis revealed that no association between IL-17A(rs2275913) gene and knee OA risk was found in Caucasian population.

**Conclusions:**

This meta-analysis revealed that the IL-17A(rs2275913) gene polymorphisms may increase the risk of knee OA in Asians, and the IL-17F(rs763780) gene polymorphisms may increase the risk of knee OA both in Asians and Caucasians. However, because of the limitations of the present study, additional larger studies are needed to confirm our findings in the future.

## Background

Knee osteoarthritis (OA) is a common joint disease which is characterized by degeneration of articular cartilage and subsequent subchondral bone changes [1]. The incidence of knee OA is high, with an estimated 14 million symptomatic patients in the USA [2]. Therefore, it is an enormous burden on the national economy and healthcare system. Knee OA is a combined consequence of environmental and genetic factors, while genetic factors account for nearly 50% of the risk of knee OA development [3]. Though the underlying mechanisms remain unknown, many studies have demonstrated that the occurrence of osteoarthritis is related to genetic factors, including interleukin-17 family [4–7].

Interleukin-17 (IL-17) families include six members (A, B, C, D, E, F), and they play active roles in autoimmune diseases and inflammatory diseases [8, 9]. IL-17A is a member of IL-17 families and is a pro-inflammatory cytokine associated with many inflammatory diseases, such as rheumatoid arthritis (RA), ankylosing spondylitis, and systemic lupus erythematosus [10]. Similarly, IL-17F earned increasing attention due to its great comparability of IL-17A. Previous studies reveal that IL-17 is expressed in synovial tissues, and could contribute to cartilage degeneration, the main cause of knee OA, by inducing the release of chemokines by chondrocytes [11]. Although knee OA is considered a non-inflammatory condition earlier, with further researches on its physiopathology, it is generally believed that immune cell infiltration and cytokine secretion are a feature of knee OA [12]. The expression of IL-17A and IL-17F has turned out to be affected by single nucleotide polymorphisms (SNPs). These polymorphisms have allele-specific effects on the regulation of IL-17 gene transcription and are associated with the development and/or progression of some common diseases [13]. Thus, we hypothesized that IL-17A(rs2275913) and IL-17F(rs763780) gene polymorphisms are associated with knee OA risk.

Up to now, several studies have investigated the relationships of IL-17A(rs2275913) and IL-17F(rs763780) polymorphism with knee OA risk in different countries and draw different conclusions [14–19]. Jiang [14] and Han [15] found that the IL-17A(rs2275913) polymorphism was involved in high risk of KOA while the IL-17F(rs763780) was not. Bai [16] and Eftedal [19] found that IL-17A/F gene polymorphisms all related to the high-risk knee OA occurrence. Bafrani [18] found that IL-17A(rs2275913) gene may be a protective factor against knee osteoarthritis while the IL-17F(rs763780) gene may be a risk factor for knee osteoarthritis. Vrgoc [17] revealed that neither IL-17A nor IL-17F were related to the knee OA. Therefore, we conducted a meta-analysis to overcome the limitations of individual studies and to search for relevance in their findings. The purpose of this study was to evaluate whether the IL-17A(rs2275913) and IL-17F(rs763780) polymorphisms are associated with the risk of knee OA.

## Materials and methods

### Literature search strategy

**Fig. 1:**
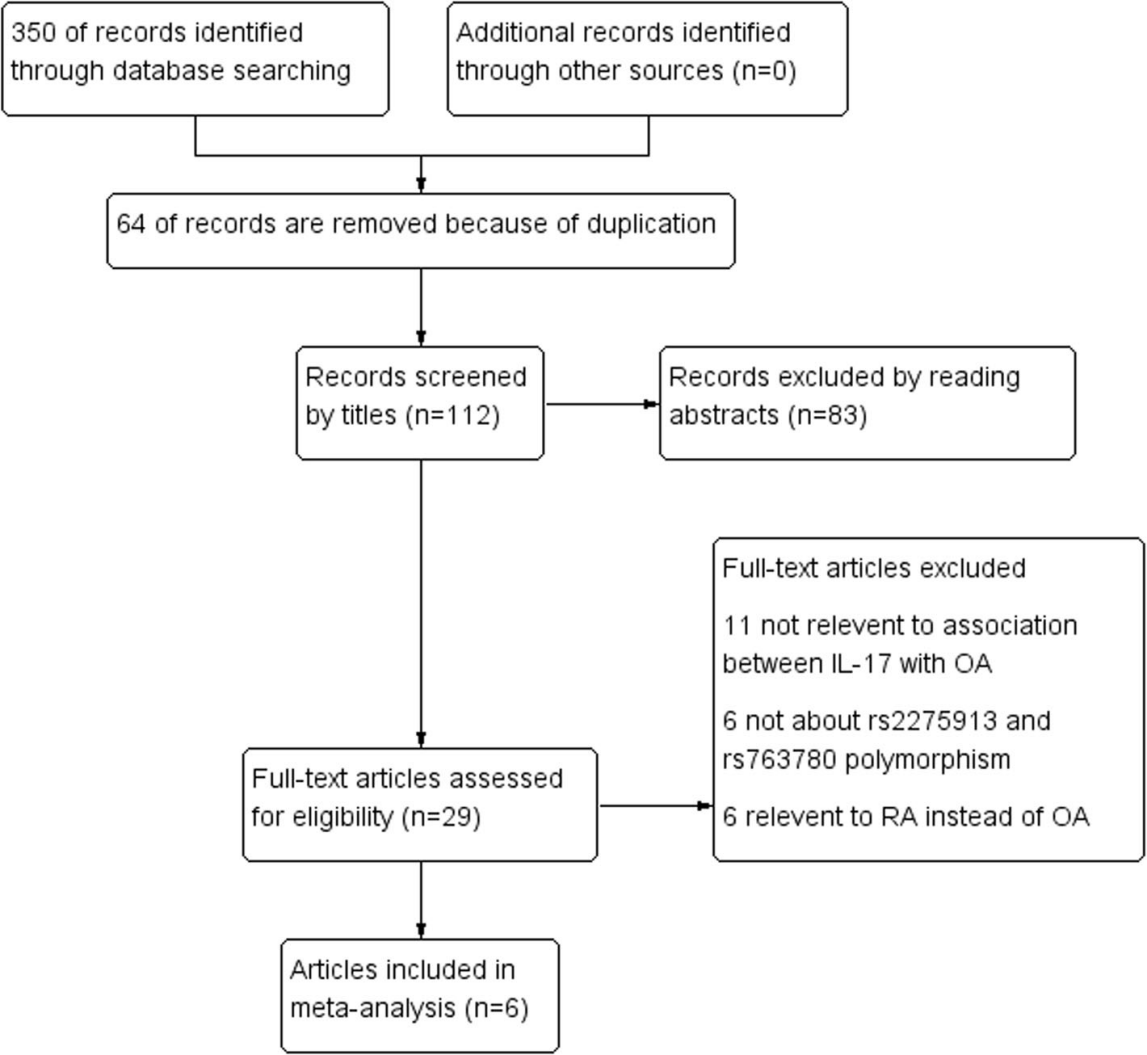
Flowchart of the literature search and selection for the present study

We conducted a comprehensive literature search using the electronic databases PubMed, Medline, Cochrane Library, Web of science, Embase, and Google Scholar to identify studies published in English (last search was updated on June 20, 2019). The search strategy was based on the following keywords: (“IL-17” OR “interleukin-17”) OR (“polymorphism” OR “variant” OR “SNP”) AND (“knee osteoarthritis” OR “KOA”). No other restrictions were placed on the search. Full text was obtained if the abstract was insufficient to allow us to include or exclude a study. Furthermore, the reference lists of all the related citations were examined to identify any initially omitted studies. All the literature searches were performed according to the Preferred Reporting Items for Systematic Reviews and Meta-Analyses (PRISMA) guidelines (Additional file 1). According to the following criteria, six studies were included in this meta-analysis (Fig. 1).

### Inclusion and exclusion criteria

Two researchers screened the relevant investigations and further determined the eligible studies which met the following inclusion criteria: (1) case-control study on humans, (2) study evaluating the association between IL-17A(rs2275913) and IL-17F(rs763780) gene polymorphisms and susceptibility to knee OA, and (3) study with sufficient genetic frequency for extraction. The exclusion criteria were as follows: (1) insufficient data on genotype or allele frequency for calculation of odds ratio (OR) and corresponding 95% confidence interval (CI); (2) animal model research, review or case report; and (3) duplicate or overlapping publication. In cases of uncertainty regarding any of the above essential information, all questionable publications were discussed and addressed by consensus. The full article was retrieved for further scrutiny, or the authors of the individual trials were contacted directly for further information when necessary.

### Data extraction

Two researchers extracted all data independently according to the criteria described above. We developed a data extraction sheet including year of publication, the first author’s name, ethnicity, source of control groups, genotype, genotyping method, and allele frequency. Any controversies of the data were discussed within our research team and the authors reached a consensus on all items.

### Study quality assessment

Study quality assessment was conducted by using the Newcastle-Ottawa Quality Assessment Scale (NOS). The quality score of each study was based on three categories: selection (4 items, 1 point each), comparability (1 item, up to 2 points), and exposure/outcome (3 items, 1 point each). Each study scored from 0 point (worst) to 9 points (best), and scored 6 or less were classified as low quality, whereas studies scoring 7 or higher were defined as high quality. The results of study quality assessment were shown in Tables 1 and 2.

**Table 1:** Characteristics of the included studies of IL-17A(rs2275913)

Authors	Years	Case			Control			Genotype	Genotyping method	Ethnics	HWE test	NOS
		GG	GA	AA	GG	GA	AA				*p* value	
Jiang [14]	2019	204	170	34	289	194	23	IL-17A rs2275913	PCR-RFLP	Asian	Y (0.180)	8
Han [15]	2014	52	109	141	97	106	97	IL-17A rs2275913	PCR-SSCP	Asian	N (0.000)	7
Bai [16]	2019	189	271	134	207	265	104	IL-17A rs2275913	PCR	Asian	Y (0.235)	7
Bafrani [18]	2019	83	35	9	69	51	7	IL-17A rs2275913	PCR-RFLP	Caucasian	Y (0.539)	8
Vrgoc [17]	2017	154	156	48	190	172	45	IL-17A rs2275913	PCR	Caucasian	Y (0.520)	7
Eftedal [19]	2019	372G and 572A			396G and 638A			IL-17A rs2275913	N/A	Caucasian	N/A	6

*P* value for Hardy-Weinberg equilibrium in the control groups. *N/A*, data not available, *Y* yes, *N* no, *NOS* Newcastle-Ottawa Quality Assessment Scale, *PCR-RFLP* polymerase chain reaction and restriction fragment length polymorphism, *SSCP* single strand conformation polymorphism

**Table 2:** Characteristics of the included studies of IL-17F(763780)

Authors	Years	Case			Control			Genotype	Genotyping method	Ethnics	HWE test	NOS
		TT	TC	CC	TT	TC	CC				*p* value	
Jiang [14]	2019	356	49	4	423	80	2	IL-17F rs763780	PCR-RFLP	Asian	Y (0.384)	8
Han [15]	2014	226	59	17	236	56	8	IL-17F rs763780	PCR-SSCP	Asian	N (0.044)	7
Bai [16]	2019	380	188	26	411	155	10	IL-17F rs763780	PCR	Asian	Y (0.287)	7
Bafrani [18]	2019	98	26	3	112	13	2	IL-17F rs763780	PCR-RFLP	Caucasian	Y (0.091)	8
Vrgoc [17]	2017	393	48	1	493	34	1	IL-17F rs763780	PCR	Caucasian	Y (0.610)	7
Eftedal [19]	2019	905 T and 39C			1004 T and 30C			IL-17F rs763780	N/A	Caucasian	N/A	6

*P* value for Hardy-Weinberg equilibrium in the control groups. *N/A* data not available, *Y* yes, *N* no, *NOS* Newcastle-Ottawa Quality Assessment Scale, *PCR-RFLP* polymerase chain reaction and restriction fragment length polymorphism, *SSCP* single strand conformation polymorphism

### Statistical analysis

Pooled odds ratios (ORs) with 95% confidence intervals (CIs) were calculated to assess the association between IL-17A(rs2275913) and IL-17F(rs763780) gene polymorphisms and knee OA susceptibility. The observed genotype frequencies in control groups of the IL-17A(rs2275913) and IL-17F(rs763780) gene polymorphisms were assessed for Hardy Weinberg equilibrium (HWE) by using the Chi-square test. Three genetic models were used: (1) allele model, (2) recessive model, and (3) dominant model. *p* < 0.05 was considered significant. Heterogeneity assumption across studies were assessed by using the *Q* statistic with its *p* value and *I*^2^ statistic. If *I*^2^ < 50% and *p* > 0.10, a fixed effects model was used in the calculations, otherwise, a random effects model was applied [20, 21]. Subgroup analysis was carried out on the basis of ethnicity. Potential publication bias was assessed with Begg’s test. Sensitivity analysis by omitting each study in turn determines the impact on the heterogeneity test and assessing the stability of the overall results. All statistical analyses were conducted in Stata15.1 (Stata Corporation, College Station, TX, USA).

## Results

### Characteristics of the included studies

The online search yielded 350 records, of which 64 duplicates were removed. Then 83 of 112 remaining records were excluded after reviewing of abstracts. The remaining 29 articles were sent for full text review, which excluded 11 articles without relevant to the association between IL-17 gene with knee OA, 6 not about rs2275913 and rs763780 polymorphism, and 6 relevant to RA instead of knee OA (Fig. 1). Finally, we identified six case-control studies about the association between IL-17 gene polymorphisms and knee OA. Our meta-analysis involved a total of 2134 knee OA patients and 2306 controls (rs2275913) and 2134 knee OA patients and 2426 controls (rs763780). The distribution of genotypes of all the studies was tested with HWE and only 1 article [15] did not conform to it. Because genotype distribution data was not reported by Eftedal [19], only allele model was analyzed in the overall population and Caucasian population. More characteristics of the included articles are shown in Tables 1 and 2.

### Meta-analysis

#### Association between rs2275913 polymorphism and knee OA susceptibility

**Fig. 2:**
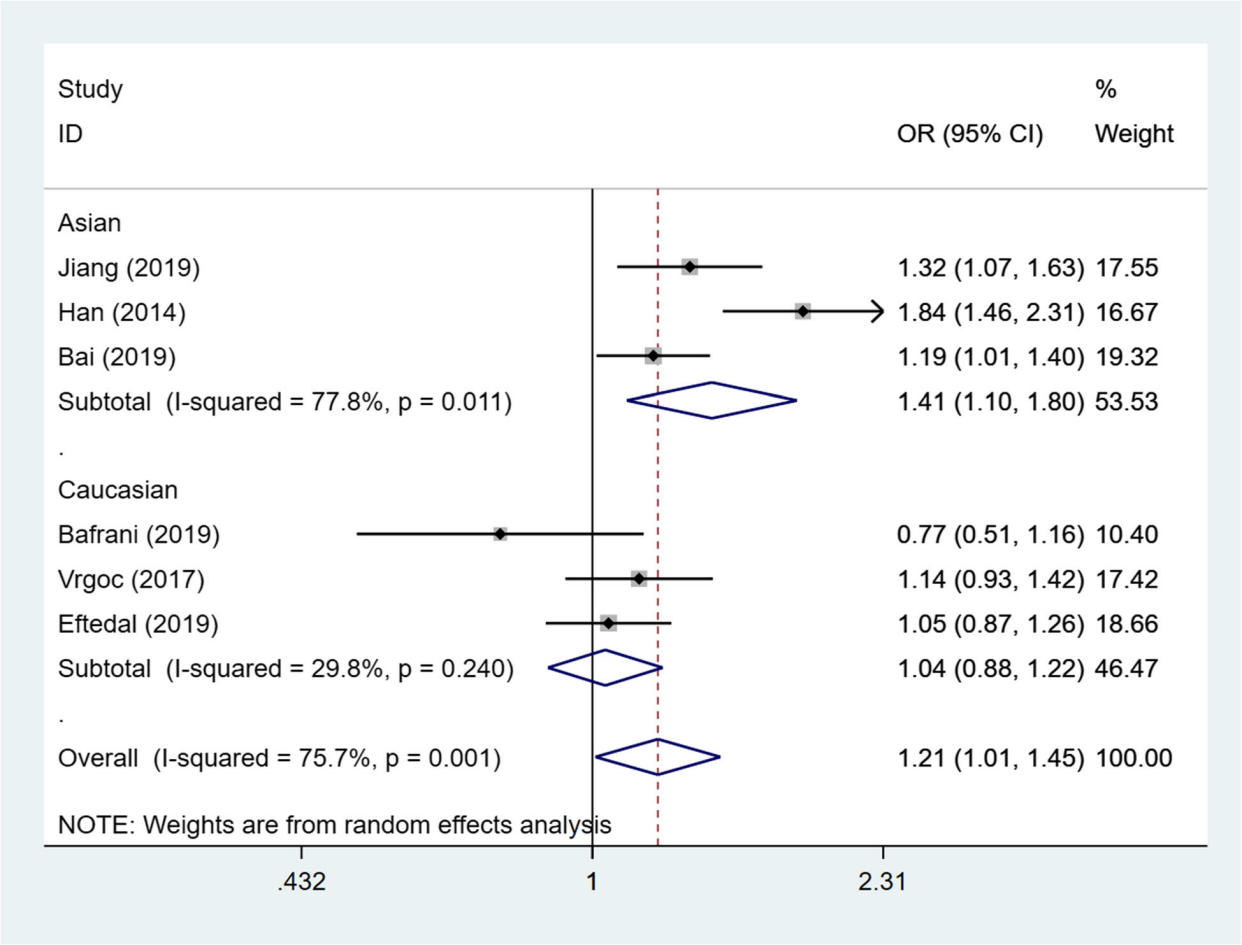
Forest plot showing OR for the associations between the IL-17A(rs2275913) gene polymorphism and knee OA risk (allele model)

**Fig. 3:**
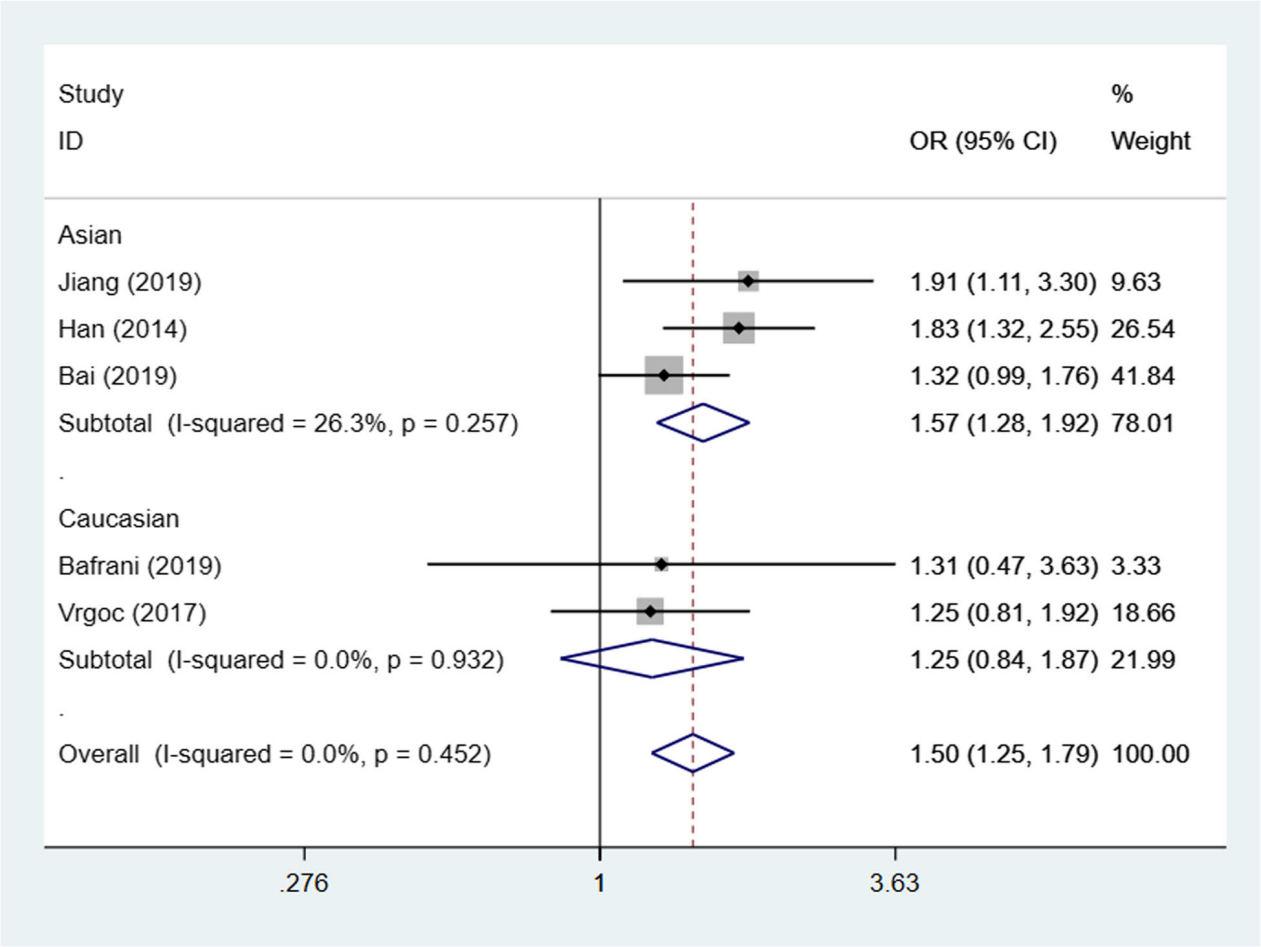
Forest plot showing OR for the associations between the IL-17A(rs2275913) gene polymorphism and knee OA risk (recessive model)

**Fig. 4:**
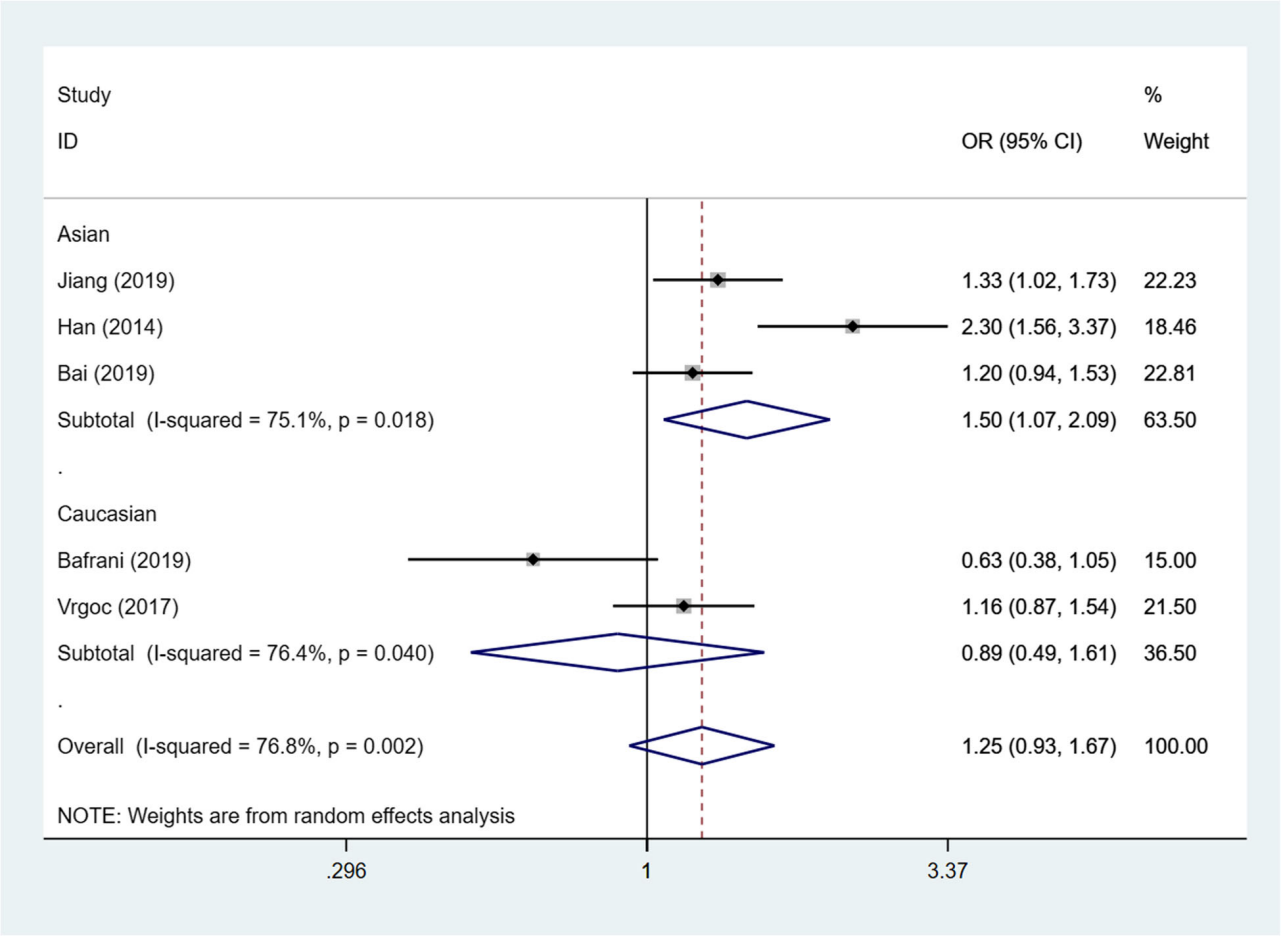
Forest plot showing OR for the associations between the IL-17A(rs2275913) gene polymorphism and knee OA risk (dominant model)

Based on the test results of heterogeneity, we used fixed effect model (*I*^2^ < 50% and *p* > 0.10) for recessive model and random effect model (*I*^2^ > 50% and *p* < 0.10) for allele model and dominant model to make this meta-analysis. The result of the association between IL-17A(rs2275913) gene polymorphisms and the risk of knee OA and the heterogeneity test are shown in Table 3. Our analysis suggested that allele A and genotype AA of the rs2275913 polymorphism were associated with high knee OA risk (allele model (A vs G) OR = 1.21, 95% CI 1.01–1.45, *p* = 0.040, recessive model (AA vs GG + GA) OR = 1.50, 95% CI 1.25–1.79, *p* = 0.000, Figs. 2, 3, and 4).

**Table 3:** OR and 95% CI for OA and IL-17A rs2275913 under different genetic models

Genetic model	Ethnics	Studies	Association test			Heterogeneity	Begg’s test *p* value
			OR	95% CI	*p* value	*p* value	
Dominant model (GA + AA vs GG)	Total	5	1.25	0.93–1.67	0.019	0.002	0.806
	Asian	3	1.50	1.07–2.09	0.693	0.018	
	Caucasian	2	0.89	0.49–1.61	0.138	0.040	
Allele model (A vs G)	Total	6	1.21	1.01–1.45	0.040	0.001	0.851
	Asian	3	1.41	1.10–1.80	0.006	0.011	
	Caucasian	3	1.04	0.88–1.22	0.658	0.240	
Recessive model (AA vs GG + GA)	Total	5	1.50	1.25–1.79	0.000	0.452	0.806
	Asian	3	1.57	1.28–1.92	0.000	0.257	
	Caucasian	2	1.25	0.84–1.87	0.265	0.932

In order to reduce the heterogeneity of the analysis, we performed sub-group analyses to investigate the effect of the ethnicity with the risk of the knee OA. The results showed that there was a significant difference between the subgroups. In the Asian group, all the three models showed a great association between IL-17A(rs2275913) gene polymorphisms and the risk of knee OA (dominant model: OR:1.50, 95% CI 1.07–2.09, allele model: OR1.41, 95% CI 1.10–1.80, recessive model: OR1.57, 95% CI 1.28–1.92). Especially in recessive model, the heterogeneity was low (*I*^2^ = 26.3%, *p* = 0.257), which makes the results more valuable. In the Caucasian group, however, the results suggested that the IL-17A(rs2275913) gene polymorphisms were not associated with knee OA risk in all genetic models (Table 3) and the heterogeneity was decreased significantly in recessive model (*I*^2^ = 0%, *p* = 0.932) and allele model (*I*^2^ = 29.8%, *p* = 0.240).

Sensitivity analysis was also used to determine the pooled ORs regarding the effects of this SNP on knee OA risk by omitting each study in turn to determine the effect on the heterogeneity test and evaluate the stability of the overall results. Furthermore, one article in this study did not conform to HWE and the investigation was excluded in our sensitivity analysis. We found that the results in our sensitivity analysis were consistent with those in non-sensitivity analysis; the results indicated that our data were stable and credible.

#### Association between rs763780 polymorphism and knee OA susceptibility

**Fig. 5:**
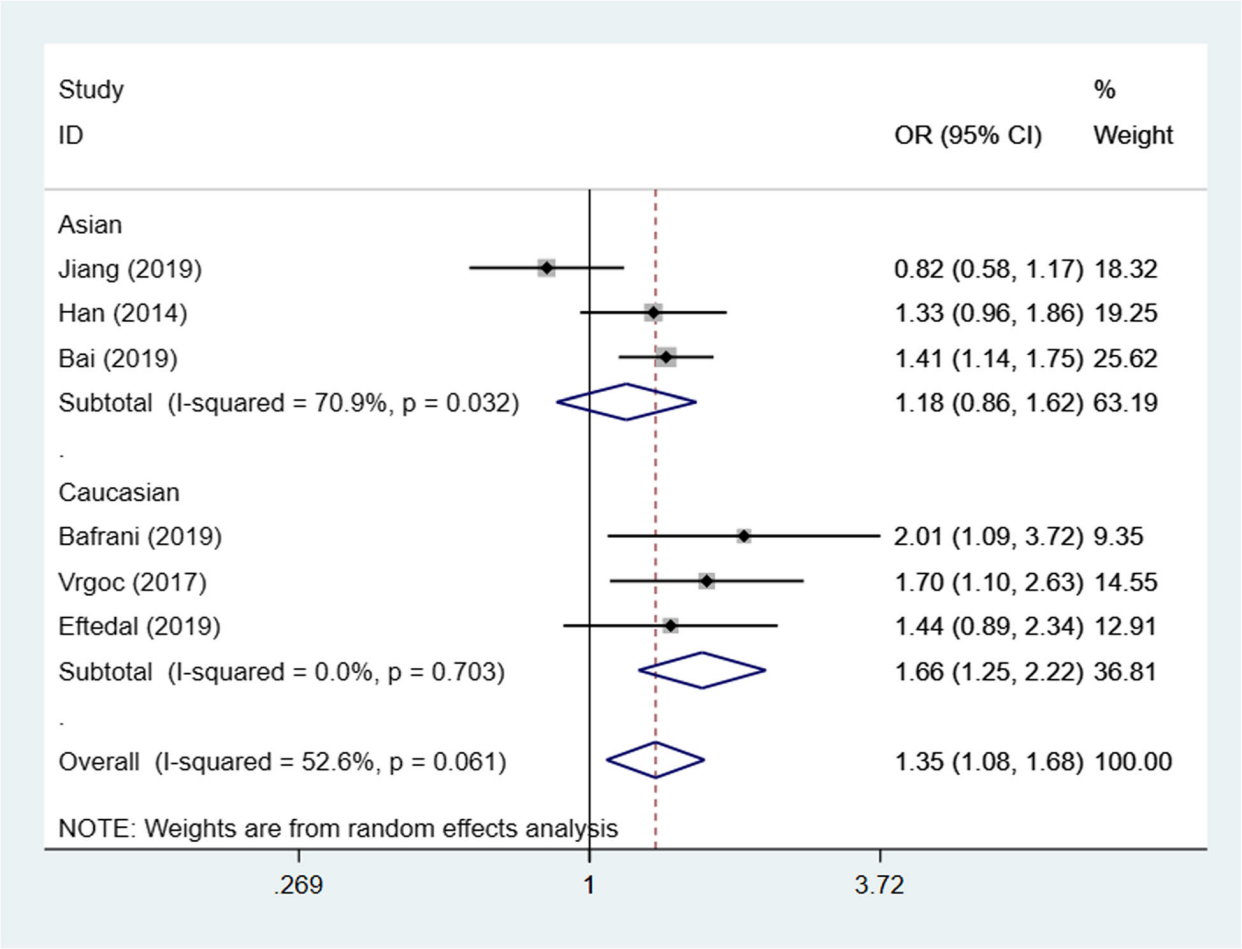
Forest plot showing OR for the associations between the IL-17F(rs763780) gene polymorphism and knee OA risk (allele model)

**Fig. 6:**
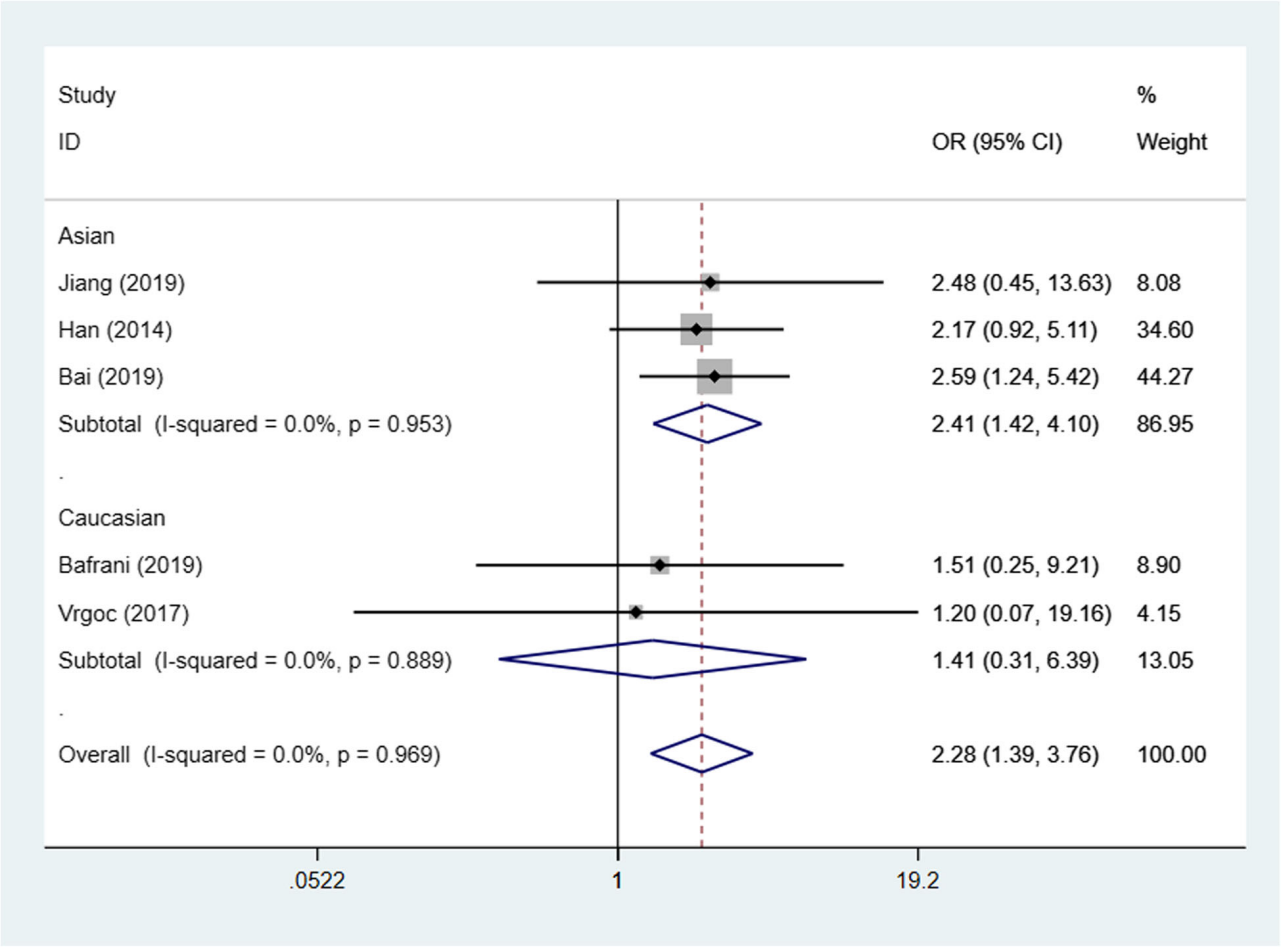
Forest plot showing OR for the associations between the IL-17F(rs763780) gene polymorphism and knee OA risk (recessive model)

**Fig. 7:**
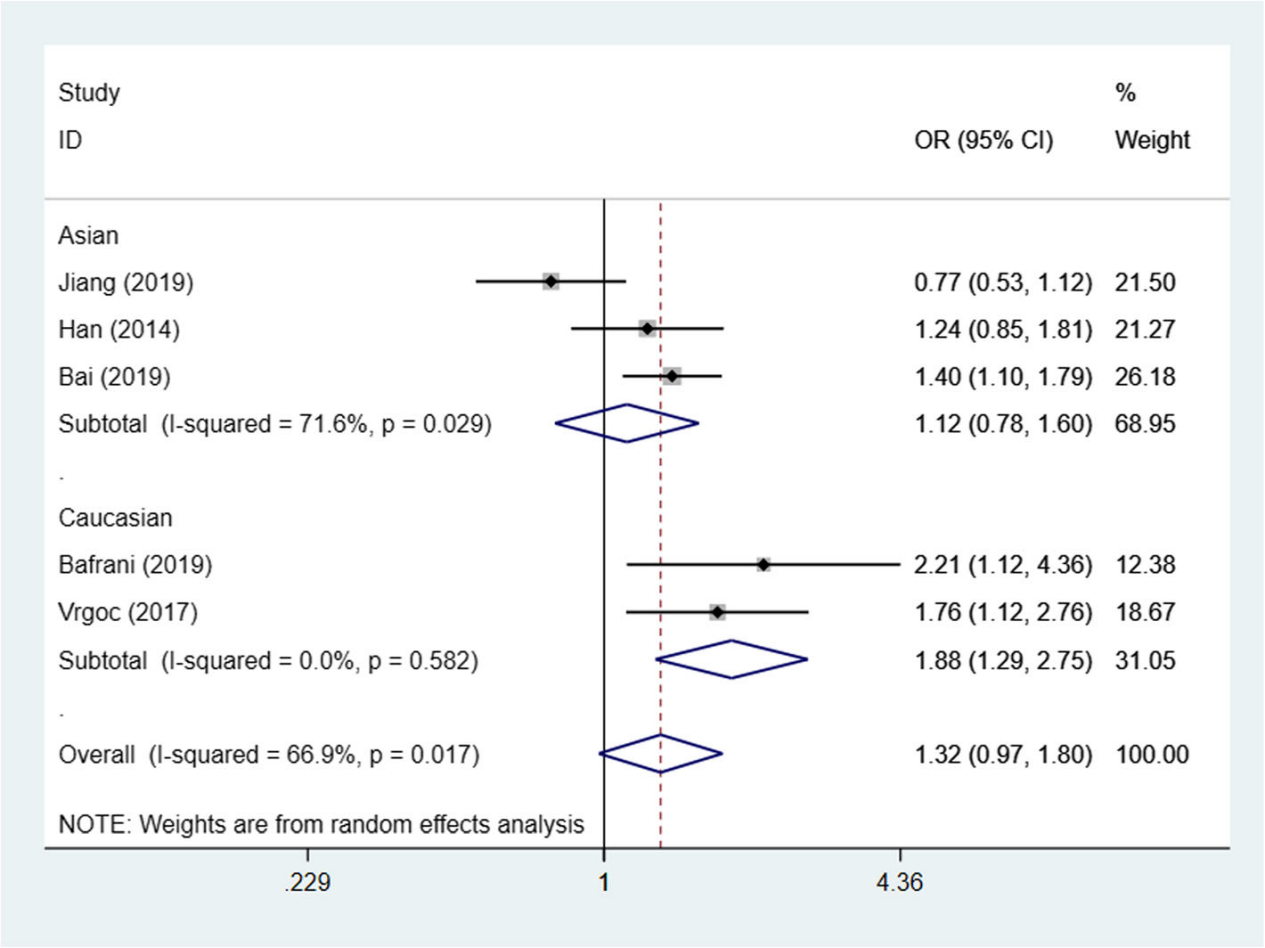
Forest plot showing OR for the associations between the IL-17F(rs763780) gene polymorphism and knee OA risk (dominant model)

Based on the test results of heterogeneity, we used fixed effect model (*I*^2^ < 50% and *p* > 0.10) for recessive model and random effect model (*I*^2^ > 50% and *p* < 0.10) for allele model and dominant model to make this meta-analysis. The results of the association between IL-17F(rs763780) gene polymorphisms and the risk of knee OA and the heterogeneity test are shown in Table 4. Our analysis suggested that the C allele and genotype CC of the rs763780 polymorphism were associated with knee OA risk (allele model (C vs T) OR = 1.35, 95% CI 1.08–1.68, *p* = 0.008, recessive model (CC vs TT + TC) OR = 2.28, 95% CI 1.39–3.76, *p* = 0.001, Figs. 5, 6, and 7).

**Table 4:** OR and 95% CI for OA and IL-17F rs763780 under different genetic models

Genetic model	Ethnics	Studies	Association test			Heterogeneity	Begg’s test *p* value
			OR	95% CI	*p* value	*p* value	
Dominant model (TC + CC vs TT)	Total	5	1.32	0.97–1.80	0.073	0.017	0.221
	Asian	3	1.12	0.78–1.60	0.533	0.029	
	Caucasian	2	1.88	1.29–2.75	0.001	0.582	
Allele model (C vs T)	Total	6	1.35	1.08–1.68	0.008	0.061	0.452
	Asian	3	1.18	0.86–1.62	0.302	0.032	
	Caucasian	3	1.66	1.25–2.22	0.001	0.703	
Recessive model (CC vs TT + TC)	Total	5	2.28	1.39–3.76	0.001	0.953	0.086
	Asian	3	2.41	1.42–4.10	0.001	0.889	
	Caucasian	2	1.41	0.31–3.76	0.655	0.969

We then performed sub-group analyses to investigate the effect of the ethnicity with the risk of the knee OA. In the Asian group, recessive model showed a great association between IL-17F(rs763780) gene polymorphisms and the risk of knee OA (recessive model: OR 2.41, 95% CI 1.42–4.10), and the heterogeneity was low (*I*^2^ = 0%, *p* = 0.953) which makes the results more valuable. In the Caucasian group, the results suggested that the IL-17F(rs763780) gene polymorphisms were associated with knee OA risk in dominant model and allele model (dominant model: OR 1.88, 95% CI 1.29–2.75; allele model: OR 1.66, 95% CI 1.25–2.22, Table 4). The heterogeneity was decreased significantly in all three models (*I*^2^ = 0%, *p* > 0.05) in the Caucasian group, we believe that ethnic was the main source of heterogeneity.

Sensitivity analysis was used to determine the pooled ORs regarding the effects of this SNP on knee OA risk by omitting each study in turn to determine the effect on the heterogeneity test. One study involved in our study did not conform to HWE and it was excluded in our sensitivity analysis. We found that the results in our sensitivity analysis were consistent with those in non-sensitivity analysis, the results indicated that our data were stable and credible.

#### Publication bias analysis

Begg’s test were performed to quantitatively evaluate the publication bias of literatures on knee OA. The results of the Begg’s test suggested that there was no evidence of publication bias in the meta-analyses of the association between the IL-17A(rs2275913) and IL-17F(rs763780) gene polymorphisms and risk of knee OA ((*p* > 0.05, Tables 3 and 4).

## Discussion

To our knowledge, this is the first meta-analysis to investigate the associations between IL-17A(rs2275913) and IL-17F(rs763780) gene SNPs and knee OA risk. The most important finding was that polymorphisms of IL-17A(rs2275913) and IL-17F(rs763780) gene associate with the risk of knee osteoarthritis in different ethnics. This finding proves our assumption that polymorphisms of IL-17A/F are associated with the occurrence of knee osteoarthritis and is a risk factor for the knee OA.

The definite mechanisms of the knee OA remain unknown, but many studies have shown that genetic factors are considered to be strong determinants with them, and the occurrence of knee osteoarthritis is related to inflammatory and cytokines [22–24]. Recent studies have suggested that knee OA might be considered a chronic inflammatory disorder, elevated levels of IL-1, IL-6, IL-17,TNF-α, and other acute-phase proteins that are found in patients with cartilage degradation [25]. A kind of conventional viewpoint is that inflammatory mechanism plays a crucial role in the pathogenesis and evolution of cartilage degeneration and expression of inflammatory reaction [26–28]. IL-17 is one of the most important regulators of innate and adaptive immune responses, and it is expressed in synovial tissues, and could contribute to cartilage degeneration and synovial infiltration in joint by inducing the release of chemokines by chondrocytes [29, 30]. Furthermore, studies in animal models suggest that IL-17 knock-out mice contribute to more severe RA than wild-type animals [31]. We have reasons to believe that IL-17 may contribute to the development of knee OA. Therefore, we conducted this meta-analysis to provide more confident evidence.

In our study, 6 eligible studies, including 2134 knee OA patients and 2306 controls for gene rs2275913 and 2134 knee OA patients and 2426 controls s for gene rs763780, were identified and analyzed. We demonstrated the association between IL-17A(rs2275913) and IL-17F(rs763780) and the knee OA risk by a meta-analysis to obtain a convincing conclusion. Our research suggests that IL-17A(rs2275913) and IL-17F(rs763780) gene polymorphisms are a risk factor for knee osteoarthritis (*p* < 0.05). Different alleles play different roles in the occurrence of knee OA. As for IL-17A(rs2275913), it is believed that allele A can promote the high expression of IL-17A, which may be a risk factor for the occurrence of osteoarthritis. In our study, the A allele in case groups was indeed higher than that in the control groups. And we found the OR was higher than 1 in allele model and recessive model, which means A allele can be a mutant gene influence the development of knee OA. The genotype of IL-17F(rs763780) was similar to IL-17 A(rs2275913). The OR of allele model (C vs T) and recessive model (CC vs TT + TC) was higher than 1, while the OR with 95% CI of dominant model (TC + CC vs TT) was 0.97–1.80. This means CC genotype can increase the risk of knee OA, and we can conclude that the C allele was a mutant gene and recessively expressed in human.

Subgroup analysis revealed that the results were altered between Asian and Caucasian groups. The heterogeneity was decreased significantly in recessive model (*I*^2^ = 0%, *p* = 0.932) and allele model (*I*^2^ = 29.8%, *p* = 0.240) of IL-17A(rs2275913) and all three models (*I*^2^ = 0%, *p* > 0.05) of IL-17 A(rs2275913) in the Caucasian group, we believe that ethnic was the main source of heterogeneity. Heterogeneity mainly exists in the Asian group, which may be related to the complex ethnic composition of the Asian population. Subgroup analysis stratified by ethnicity demonstrated that A allele increased the risk of knee OA in Asian. While in Caucasian, no association was found between IL-17 A(rs2275913) and the risk of knee OA. The results seem intriguing that there is an association in the overall population but without positive result in subgroups. It could be probably statistically insufficient when the majority of cases of the study were originated from Asian. And one other possibility may relate to the different expression patterns of IL-17A and IL-17F in non-homogeneous ethnic populations.

Despite the fact that the result shows a relevance between IL-17A(rs2275913) and IL-17F(rs763780) and risk of knee OA, there are still some facts we cannot ignore. Firstly, the occurrence of knee osteoarthritis is a comprehensive result of a variety of factors and genes, and a single gene may have little impact on this result. Secondly, there may be some unidentified alleles affecting the expression of IL-17, and the gene loci (rs2275913, rs763780) may not be the only factor affecting the expression of IL-17 in the population. Third, regardless of the fact that IL-17A and IL-17F can mediate cartilage degeneration, it may not be a direct determinant of osteoarthritis. Many cytokines of IL-17 family are involved in the inflammatory reaction. The destruction of articular cartilage is the common result of many cytokines. IL-17 cannot directly reflect the effect of inflammatory reaction changes on osteoarthritis. Subgroup analysis in our study shows no statistical difference in different ethnic groups; gene expression may still have a certain influence, in spite of considering the limited articles involved.

There are still some limitations in our paper. Firstly, our several genetic models have great heterogeneity when combining OR values, which may affect the accuracy of the article. For this reason, we used random effect model to combine OR values and conduct subgroup analysis to reduce the impact of heterogeneity. In this way, results of heterogeneity test are acceptable in some subgroups. Secondly, although six papers have been included in the study, the data scale is still small, and further experiments with larger samples in different ethnic populations are needed to verify this conclusion. Thirdly, since the data is not completely available, the potential confounding factors (such as age, gender) were not adjusted in this study. Last, though there was no obvious publication bias revealed by Begg’s test, the selection bias could not be completely removed because only studies published in English were included.

## Conclusion

In conclusion, this meta-analysis revealed an association between the polymorphism of IL-17A(rs2275913) gene and high risk of knee OA only in Asians. By contrast, the IL-17F(rs763780) gene polymorphisms may increase the risk of knee OA both in Asians and Caucasians. However, because of the limitations of the sample size, further studies with larger samples in different ethnic populations could be performed to verify this conclusion.

## Supplementary information

**Additional file 1:** PRISMA 2009 Checklist.

## Data Availability

Not applicable.

## Abbreviations

IL: Interleukin
CIs: Confidence intervals
OR: Odds ratio
OA: Osteoarthritis
SNPs: Single nucleotide polymorphisms
HWE: Hardy Weinberg equilibrium
